# Editorial: Phenylpropanoid biosynthesis in plants

**DOI:** 10.3389/fpls.2023.1230664

**Published:** 2023-06-16

**Authors:** Md Abdur Rahim, Xuebin Zhang, Nicola Busatto

**Affiliations:** ^1^ Department of Genetics and Plant Breeding, Sher-e-Bangla Agricultural University, Dhaka, Bangladesh; ^2^ State Key Laboratory of Crop Stress Adaptation and Improvement, Henan Joint International Laboratory for Crop Multi-Omics Research, School of Life Sciences, Henan University, Kaifeng, China; ^3^ Department of Genomics and Biology of Fruit Crops, Research and Innovation Centre, Fondazione Edmund Mach, Trento, Italy

**Keywords:** phenylpropanoid, flavonoid, anthocyanin, lignin, coumarin, biosynthesis, MYB TF, biosynthetic genes

Phenylpropanoids are bioactive secondary metabolites biosynthesized by plants from the essential amino acid phenylalanine catalyzed by the enzyme PAL (phenylalanine ammonia-lyase), which is part of the shikimate pathway. This metabolic route is crucial for the biosynthesis of various compounds, including flavonoids (anthocyanin, proanthocyanidin, flavonols etc.), lignins, coumarins, and lignans (Fraser and Chapple, 2011). Phenylpropanoids play significant roles in plant growth, development as well as responses to environmental stimuli such as tolerance and resistance against abiotic and biotic stresses. Additionally, they contribute to plant reproduction and singaling by accumulation of flavonol and anthocyanin pigments in floral parts. Moreover, several phenylpropanoid compounds are well known to act as powerful antioxidant with which greatly benefit human health. This Research Topic encompasses three original research articles and one review article focusing on phenylpropanoids biosynthesis and their molecular regulation in plants.

Yang et al. reviewed the regulations of anthocyanin-promoting R2R3-MYB TFs at various levels. The MYB TFs have been considered as the key regulator of structural genes of anthocyanin biosynthetic pathway (Rahim et al., 2014). The transcriptional regulation of R2R3-MYB TFs controlled by formation of a complex ‘MYB-bHLH-WD40’ (MBW) (Petroni and Tonelli, 2011). Moreover, their regulation also has been controlled by the upstream regulation *via* several TFs (bZIP, WRKY, NAC, ERF/AP2, HD-ZIP), natural variations in the promoter, and epigenetic regulations. The upstream TFs directly bind the promoter and regulate the transcript levels of MYBs, while the some TFs regulate indirectly through interacting with other upstream TFs. The colour diversity of different organs of important agricultural crops are mainly due to mutations (insertion, deletion, single nucleotide polymorphism) either in the coding region of the R2R3-MYB TFs leading to a nonfunctional, truncated protein or in the promoter region. For example, a 487-bp deletion in the promoter region of the MYB gene (*PpMYB10.1*) alter the flesh colour around the stone in peach fruit (Guo et al., 2020). Further, the epigenetic changes like DNA methylation in the promoter region of MYB TF gene can also induce anthocyanin pigmentation in plants (Qian et al., 2014; Jiang et al., 2020). Besides, the regulation of MYB controlled by protein-protein interaction, posttranscriptional modifications, Ubiquitination by E3 ligases, various signaling pathways (light, temperature, sugar and hormones).

Samkumar et al. studied the influence of light quality on flavonoid biosynthesis of non-climacteric bilberry (wild) at fruit ripening with detached berries and attached berries (natural on tree) technique. They have shown that red and blue light resulted differential phenolics accumulation in the fruit. The fruit berries treated with red light elevated the level of delphinidin component anthocyanins, while blue light improved other components of anthocyanin together with delphinidins in attached and detached conditions, respectively. Furthermore, the red and far-red light also increased the level of flavonols (particularly quercetin and myricetin glycoside) in both ripening situations. On the other hand, the blue light stimulated the utmost anthocyanins accumulation in detached fruit, even though red light increase anthocyanin synthesis in attached fruit. They also showed that anthocyanin compositions were uniformly distributed in detached fruits. Moreover, the expression of key flavonoid biosynthetic genes (*VmUFGT* and *VmANS*) were upregulated by both red and blue light treatments in both detached fruit berries during fruit ripening. The expression of such key regulatory gene, *VmMYBA1*, was also upregulated in detached fruit treated with blue light compared to treated one. The result of the study could be utilized for cultivation of bilberry fruit to enhance anthocyanin levels in the fruit berries.

Song et al. investigated the molecular regulation underlying the decrease in coumarin content during the bolting stage of *Peucedanum praeruptorum*. The transition between bolting and non-bolting has always been crucial for harvesting this plant, which is widely used in traditional Chinese medicine due to its high levels of furano- and dihydropyrano coumarins. The authors examined the coumarin component concentrations in both bolted and unbolted *P. praeruptorum*, focusing on praeruptorin A, praeruptorin B, praeruptorin E, peucedanocoumarin I, and peucedanocoumarin II. Their findings were complemented by a comparative co-expression analysis involving 1,573 differentially expressed genes. The results revealed a significant increase in praeruptorin A, praeruptorin B, and praeruptorin E in the bolted *P. praeruptorum*, while peucedanocoumarin I and peucedanocoumarin II showed slight increases. Additionally, the essential genes involved in coumarin biosynthesis exhibited an overall downward trend after bolting. Three *peroxidases* (*PRX*s) responsible for lignin monomer production were found to be downregulated. *PAL*, *C4H*, *HCT*, *COMT*, *CCoAOMT*, and some ABC transporters were markedly downregulated during the bolting stage. These findings suggest that the downregulation of coumarin biosynthetic genes in bolted P*. praeruptorum* ultimately leads to a reduction in coumarin formation.

In their study, Rahman et al. examined the role of Na^+^/H^+^ exchangers and Na^+^/K^+^ transporter genes in mediating salt stress responses in alfalfa (*Medicago sativa* L.), focusing on Na+ homeostasis regulation and its association with lignin biosynthesis genes. They compared two genotypes with different salt tolerance, namely Xingjiang Daye (XJD; sensitive genotype) and Zhongmu (ZM; tolerant genotype). The results showed that XJD exhibited a significant reduction in morphophysiological indices and increased oxidative stress indicators in response to Na+ accumulation. In contrast, the ZM genotype displayed a higher expression of genes associated with salt tolerance, including *SOS1* (salt overly sensitive 1), *NHX1* (sodium/hydrogen exchanger 1), and *HKT1* (high-affinity potassium transporter 1). These findings were consistent with the observed increase in lignin accumulation in the ZM genotype under stress conditions. The upregulation of several genes involved in lignin synthesis (*4CL2*, *CCoAOMT*, *COMT*, *CCR*, *C4H*, *PAL1*, and *PRX*) supported the enhanced lignin production in ZM. Moreover, ZM exhibited elevated antioxidant enzyme activity, providing enhanced defense against oxidative damage in the presence of high Na^+^ concentrations.

These articles highlight the phenylpropanoid biosynthesis, transcriptional regulation and influence of red and blue light on flavonoid biosynthesis, their role in abiotic stress (salt) and provide basic knowledge for phenylpropanoid biosynthesis for human health and stress tolerance/resistance in plants.

## Author contributions

All authors listed have made a substantial, direct, and intellectual contribution to the work and approved it for publication.
